# Prognostic Implications of a Modified Seattle Heart Failure Model Score Following Transcatheter Aortic Valve Replacement

**DOI:** 10.3390/jcm10245807

**Published:** 2021-12-11

**Authors:** Teruhiko Imamura, Nikhil Narang, Hiroshi Onoda, Shuhei Tanaka, Ryuichi Ushijima, Mitsuo Sobajima, Nobuyuki Fukuda, Hiroshi Ueno, Koichiro Kinugawa

**Affiliations:** 1 The Second Department of Internal Medicine, University of Toyama, Toyama 9300194, Japan; ohiro0203@gmail.com (H.O.); stanaka@med.u-toyama.ac.jp (S.T.); ryuryu0702@gmail.com (R.U.); soba1126@yahoo.co.jp (M.S.); nfukuda@med.u-toyama.ac.jp (N.F.); hueno@med.u-toyama.ac.jp (H.U.); kinugawa-tky@umin.ac.jp (K.K.); 2 Advocate Christ Medical Center, Oak Lawn, IL 60453, USA; nikhil.narang@gmail.com

**Keywords:** heart failure, hemodynamics, prognosis, aortic valve disease

## Abstract

Background: The Seattle heart failure model (SHFM) score is a well-known risk predictor of mortality in patients with heart failure. We validated this score in patients receiving transcatheter aortic valve replacement (TAVR) and aimed to generate further risk discrimination by adding invasive hemodynamics parameters. Methods: Patients who underwent TAVR at our institute between 2015 and 2020 were included and followed for 2 years from index discharge. Patients were randomly assigned to the derivation cohort or the validation cohort. In the derivation cohort, the original SHFM score was modified by adding baseline hemodynamics parameters to evaluate the primary outcomes: 2-year incidence of mortality or readmission from heart failure. The model performance of the modified SHFM score was evaluated in the validation cohort. Results: A total of 217 patients (median age: 86 (83, 88) years old, 64 (29%) men) were included. From the derivation cohort (N = 108), a novel modified SHFM score was constructed: 6 × (original SHFM score < 88.1%) + 5 × (pulmonary capillary wedge pressure > 14 mmHg) + 4 × (cardiac index < 2.26 L/min/m^2^), which had an improved discrimination compared with the original model (area under the curve: 0.887 vs. 0.679, *p* = 0.014). In the validation cohort (*N* = 109), the modified SHFM score showed accurate predictive discrimination of the 2-year cumulative incidence of the primary endpoint into three groups (a low score group with 0–5 points, 3%; an intermediate score group with 6–10 points, 12%; and a high score group with 11–15 points, 43%, *p* < 0.001). Conclusion: A modified SHFM score consisting of the original SHFM score and invasive hemodynamics parameters predicted mortality and morbidity following TAVR. Evaluation of the external validity of this score in other cohorts needs further investigation.

## 1. Background

With improvements in peri-procedural management, more sophisticated device technology, and identification of optimal patients, survival following transcatheter aortic valve replacement (TAVR) has increased considerably in patients with severe aortic stenosis [1,2]. However, post-TAVR readmissions due to heart failure continue to occur [3]. Several risk factors have been proposed to predict post-TAVR mortality and morbidity [4], though accurate models that reliably discriminate the risk of clinical events are lacking.

The Seattle heart failure model (SHFM) score, which is derived from several variables including demographics, laboratory, and medication data, has been shown to reliably predict clinical event rates in heart failure patients [5]. The applicability of this score has been externally validated in other clinical situations, including those with ventricular assist devices [6]. Recently, the score was revised to be more suitable for the Japanese cohort. The model might be a promising tool to further risk stratify post-TAVR clinical outcomes.

In this study, we investigated the prognostic implication of the original SHFM score in patients receiving TAVR and attempted to further modify the score by adding hemodynamics parameters, with further evaluation of the external validity in this cohort.

## 2. Methods

### 2.1. Patient Selection

We analyzed a retrospective cohort of patients who underwent TAVR at our institute between 2015 and 2020. All patients were followed from index discharge for 2 years or until July 2021. Written informed consent was obtained from all participants on admission. The institutional review board approved the study.

### 2.2. TAVR Procedure

Patients with severe aortic stenosis with max velocity > 4.0 m/s, mean pressure gradient > 40 mmHg, or aortic valve area < 1.0 cm^2^ were considered to receive TAVR by the multidisciplinary heart-valve team. Patients with low-flow low-gradient aortic stenosis were also considered to receive TAVR by performing dobutamine stress echocardiography.

TAVR was performed according to a standard protocolized approach. Patients received self-expandable valves or balloon-expandable valves via a trans-femoral, trans-subclavian, or direct aorta approach under general or local anesthesia support.

### 2.3. Clinical Variables

Demographics, laboratory, echocardiographic, hemodynamics, and medication data within one week before TAVR were collected. According to pre-TAVR baseline characteristics including demographics, laboratory, and medication data, the original SHFM score was retrospectively calculated for all patients using the website (https://jcvsd.org/WET2_SHFM/WET2_SHFM, accessed on 20 November 2021) [5].

### 2.4. Clinical Outcomes

All patients were followed at our institute or affiliated institutes. All-cause death and heart failure readmission that required IV diuretics or any other intensive therapies under in-hospital observation were defined as the primary endpoints.

### 2.5. Study Protocol

**Figure 1:**
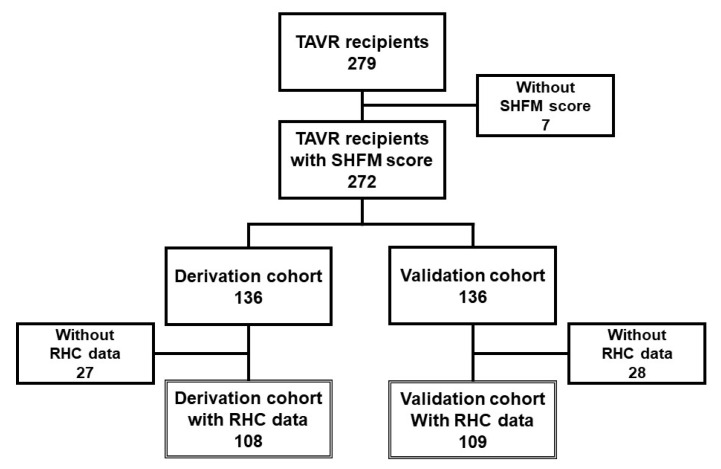
Patient selection. TAVR, transcatheter aortic valve replacement; SHFM, Seattle Heart failure Model; RHC, right heart catheterization.

All patients were randomly assigned to the derivation cohort or the validation cohort (Figure 1). Using the original SHMF score and additional hemodynamics parameters statistically chosen to best predict the primary endpoint (all-cause death or heart failure readmission) in the derivation cohort, a newly modified SHFM score was derived. The external validity of the modified SHFM score was tested in the validation cohort.

### 2.6. Statistical Analysis

We assumed all continuous variables to be non-parametric parameters irrespective of the normality of their distribution given a moderate sample size. Continuous variables are stated as the median and interquartile range and were compared using the Mann–Whitney U test. Categorical variables are stated as numbers and percentages and were compared using Fisher’s exact test. A value of 2-tailed *p* < 0.05 was considered statistically significant. Statistical analyses were performed using SPSS Statistics 22 (SPSS Inc., Armonk, IL, USA).

The primary endpoint was two-year all-cause death or heart failure readmission. Time-dependent receiver operating characteristic analysis was conducted to calculate the cutoff of each variable. In the derivation cohort, univariate and multivariate Cox proportional hazard ratio regression analyses were performed to investigate the impact of original SHFM score and hemodynamics variables on the primary endpoint. Continuous variables with *p* < 0.10 in the univariate analyses were dichotomized using cutoffs calculated in the receiver operating characteristics analyses. Dichotomized variables with *p* < 0.05 in the univariate analyses were enrolled into the multivariate analysis. A modified SHFM score was derived according to the results of the multivariate analysis and the hazard ratio of each variable.

In the validation cohort, Kaplan–Meier analysis with a log-rank test was performed to compare the cumulative incidence of the primary endpoints among the groups stratified into 3 groups by the modified SHFM score (a low score group, an intermediate score group, and a high score group). Heart failure readmission rates were compared among the 3 groups using binomial regression analyses.

## 3. Results

### 3.1. Baseline Characteristics

A total of 279 patients who received TAVR were screened for study inclusion (Figure 1). Seven patients were excluded given missing variables needed to calculate the SHFM score. Patients were randomly assigned to 136 patients in the derivation cohort and 136 patients in the validation cohort. Patients without hemodynamic data were excluded, and, finally, 108 patients in the derivation cohort and 109 patients in the validation cohort were included.

The median age was 86 (83, 88), and 64 patients were men. The peak velocity at the aortic valve was 4.5 (4.0, 4.9) msec. There were no significant differences in the baseline characteristics between the two cohorts (*p* > 0.05 for all; Table 1).

### 3.2. Derivation of Modified SHFM Score

During 730 (638, 730) days of the observational period, seven patients died and six patients had heart failure readmissions.

In the univariate analyses, the original SHFM score trended towards though did not reach statistical significance in association with the primary endpoint (*p* = 0.098; Table 2). Among the hemodynamic data, pulmonary capillary wedge pressure (PCWP) trended towards an association while cardiac index was significantly associated with the primary endpoint (*p* = 0.053 and *p* = 0.001, respectively). According to the calculated cutoff and hazard ratio obtained in the multivariate analysis, a modified SHFM score was derived: 6 × (original SHFM score < 88.1%) + 5 × (PCWP > 14 mmHg) + 4 × (cardia index < 2.26 L/min/m^2^).

**Figure 2:**
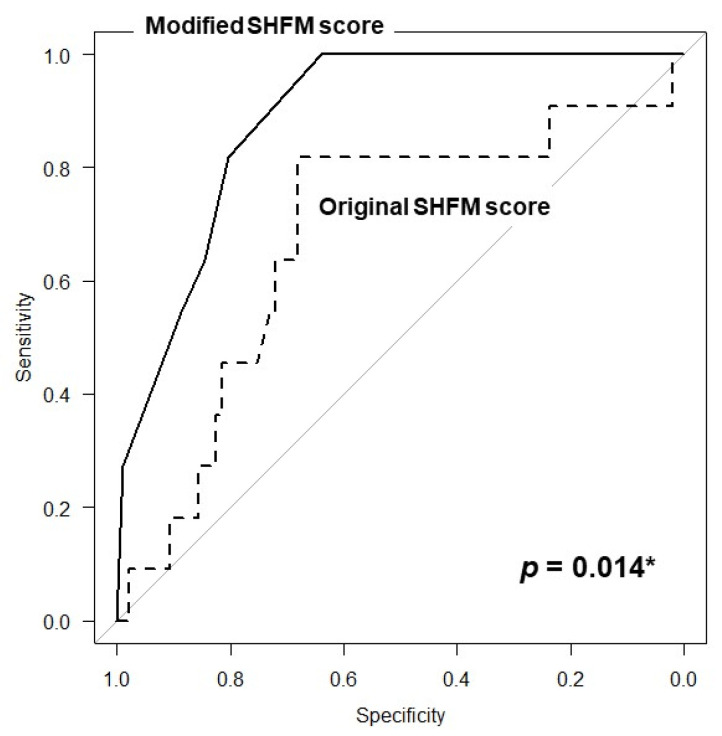
Receiver operating characteristics analyses comparing between the modified SHFM score and the original SHFM score in the derivation cohort. * *p* < 0.05.

The hazard ratio of the modified SHFM score was 1.38 (95% confidence interval: 1.18–1.61). The area under the curve of the modified SHFM score was also greater than that of the original SHFM score alone (0.887 versus 0.679, *p* = 0.014; Figure 2).

### 3.3. Validation of the Modified SHFM Score

**Figure 3:**
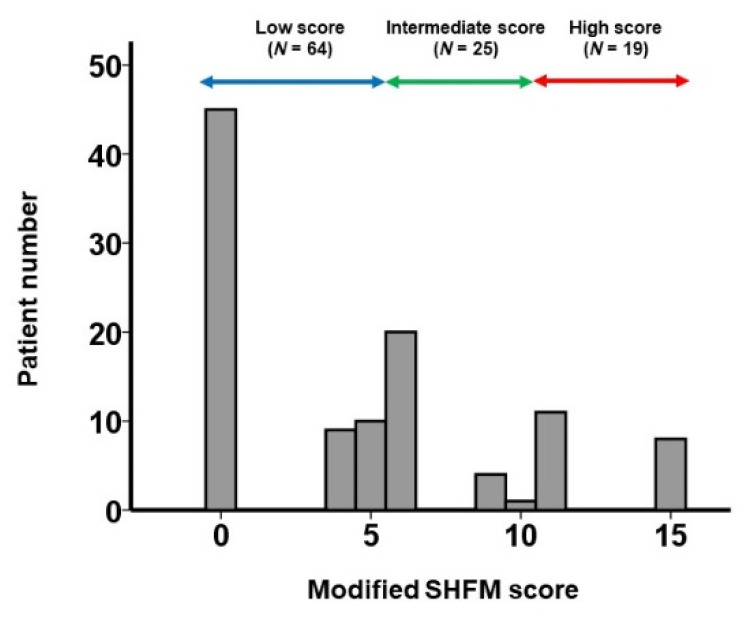
Distribution of modified SHFM score in the validation cohort. Patients were assigned to the low score group (0–5 points), the intermediate score group (6–10 points), and the high score group (11–15 points).

The modified SHFM score was calculated in all patients in the validation cohort. The distribution of the modified SHFM score in the validation cohort (*N* = 109) is shown in Figure 3. During a median of 730 (659, 730) days in the observational period, seven patients died and eight patients had heart failure readmissions.

**Figure 4:**
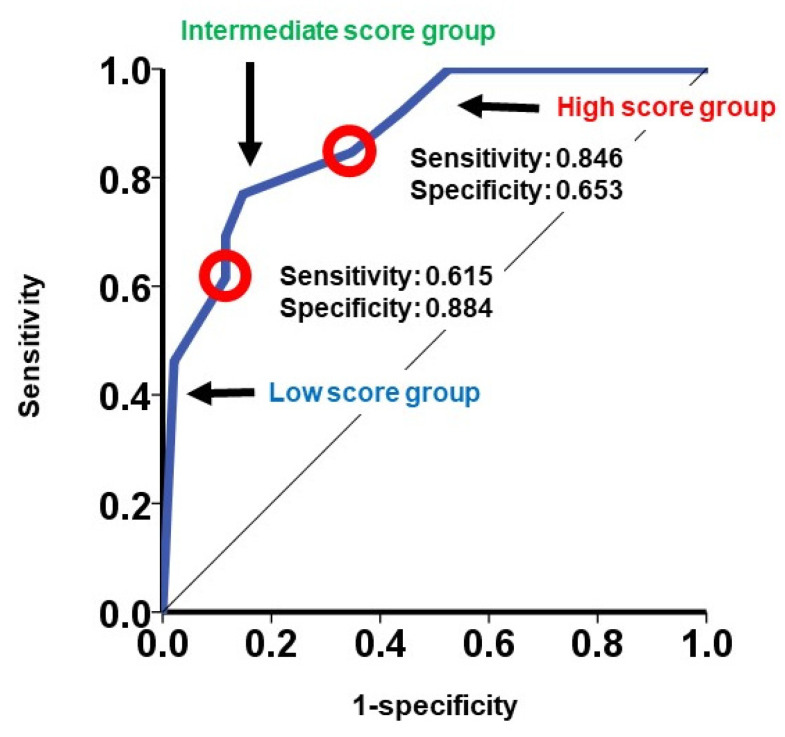
Receiver operating characteristics analysis of the modified SHFM score to predict the primary endpoint in the validation cohort.

The hazard ratio of the modified SHFM score for the primary endpoint was 1.34 (95% confidence interval: 1.18–1.52). The area under the curve of the modified SHFM was 0.879 (Figure 4).

**Figure 5:**
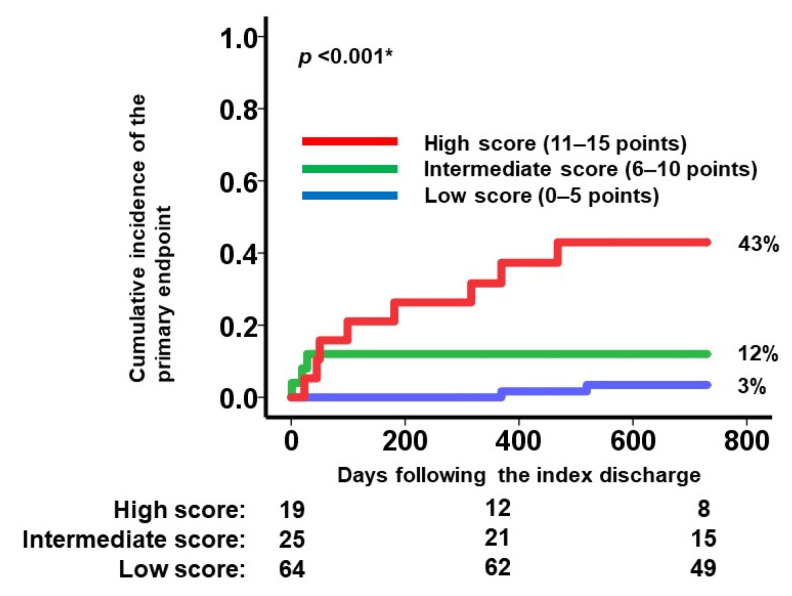
Two-year cumulative incidence of the primary endpoint stratified by the modified SHFM score. Patients assigned to the high score group (11–15 points) had a higher cumulative incidence compared with the other two groups. * *p* < 0.05 by log-rank test.

**Figure 6:**
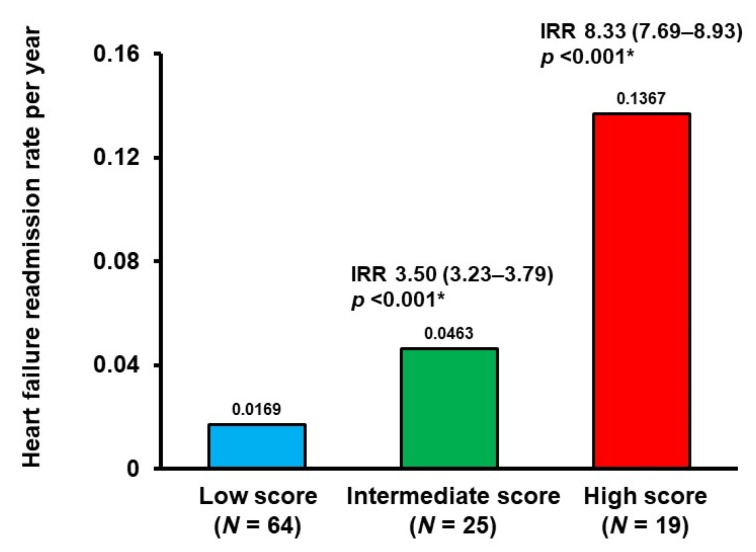
Heart failure readmission rates stratified by the modified SHFM score. Patients assigned to the low score group (0–5 points) had a lower cumulative incidence compared with the other two groups, respectively. * *p* < 0.05 compared to the low score group using negative binomial regression analyses. IRR, incidence rate ratio.

If patients were assigned to the low score group (0–5 points; *N* = 64), the intermediate score group (6–10 points; *N* = 25), or the high score group (11–15 points; *N* = 19), the 2-year cumulative incidence of the primary endpoint was stratified by these three groups (3%, 12%, and 43%, respectively; *p* < 0.001; Figure 5). The hazard ratio of the intermediate score group versus the low score group was 6.31 (95% confidence interval: 1.60–24.9, *p* = 0.009). The hazard ratio of the high score group versus the low score group was 17.6 (95% confidence interval: 3.72–83.1, *p* < 0.001). Heart failure readmission rates similarly increased in incidence when stratified by the three groups (0.0169, 0.0463, and 0.1367 events per year, respectively; Figure 6).

## 4. Discussion

In this study, we investigated the prognostic impact of the original SHFM score and further derived a modified SHFM score by adding hemodynamic data in patients undergoing TAVR for severe aortic stenosis. In the derivation cohort, we derived a modified SHFM score by adding PCWP and cardiac index data, which had superior predictability compared with the original score. In the validation cohort, the modified SHFM score also accurately stratified the 2-year cumulative incidence of the primary endpoint into three groups: a low score group, an intermediate score group, and a high score group.

### 4.1. SHFM Score

The SHFM score was originally derived from randomized control trials [5] and is designed for use in ambulatory patients with chronic heart failure, with accurate risk prediction of high-risk subsets that may benefit from advanced therapies, including durable ventricular assist devices and heart transplantation [6].

The applicability of the original SHFM score to non-Western in-hospital patients was recently validated with varying accuracy depending on the type of chronic heart failure [7]. Predictability was acceptable in patients with systolic heart failure and modest in patients with diastolic heart failure, possibly due to the lack of predictive covariates more relevant to patients with diastolic heart failure.

In this study, the original SHFM score was moderately risk-discriminative in predicting death or heart failure readmissions in patients with severe aortic stenosis undergoing TAVR (area under the curve: 0.679).

### 4.2. Modified SHFM Score

The predictive accuracy of the original SHFM score was further improved by the addition of hemodynamics parameters. The original SHFM score did not include invasive hemodynamic data [5], which made this score unique with the addition of alternative parameters that commonly predict risk in patients with chronic heart failure [8].

Our practice is to routinely perform a right heart catheterization before cardiac interventions including TAVR to better optimize a patient in a tailored fashion in hopes of mitigating downstream clinical risks related to heart failure [9]. Considering this, this modified score may be used in centers with similar practices and capabilities to perform routine hemodynamic assessments.

In this study, both elevated PCWP and lower cardiac index were independently associated with the primary endpoint, possibly due to diastolic dysfunction, which increases left atrial pressure and smaller ventricular cavities comprising stroke volume, both of which are common in patients with severe aortic stenosis. Furthermore, other prior studies have also reported hemodynamic derangements, including from valvular disease including severe mitral and tricuspid regurgitation and elevated plasma volume, to be independent predictors of morbidity and mortality following TAVR [10,11,12,13].

### 4.3. Clinical Implications of the Modified SHFM Score

The modified SHFM score would be particularly useful as part of shared decision-making for TAVR candidates [14]. In patients who have intrinsic severe diastolic dysfunction that may remain post-TAVR despite valve correction, the estimated residual risk that remains must be considered in light of advanced age or other comorbid conditions that may independently negate a long-term benefit of TAVR. Considering these additive risks as shown in our modified SHFM score, alternative therapies including medication adjustment, palliative care, and balloon aortic valvuloplasty alone might be considered, instead of the standard TAVR procedure [15].

Given our findings, aggressive interventions to optimize hemodynamics before TAVR might improve the score and clinical outcomes, although further prospective analyses of this unique question are needed.

### 4.4. Limitations

This is a retrospective study comprised of a moderate sample size. We observed an association between modified SHFM score and clinical outcomes following TAVR, though causality given the observational nature of this study remains uncertain. We confirmed the predictability of the modified SHFM score in our validation cohort, but the applicability of this score to other external cohorts would require detailed validation analyses. The modified SHFM score was divided into three groups (low, intermediate, and high score) according to the findings of receiver operating characteristics analyses. Further optimal groupings that well stratify the patients’ risks might exist.

## 5. Conclusions

A modified SHFM score, consisting of the original SHFM score and two hemodynamics parameters, was associated with morbidity and mortality following TAVR.

## Figures and Tables

**Table 1:** Baseline characteristics.

	Total (*N* = 217)	Derivation Cohort (*N* = 108)	Validation Cohort (*N* = 109)	*p* Value
Demographics				
Age, years	86 (83, 88)	86 (83, 88)	85 (82, 88)	0.56
Men	64 (29%)	33 (31%)	31 (28%)	0.49
Body surface area, m^2^	1.38 (1.28, 1.52)	1.38 (1.26, 1.49)	1.39 (1.30, 1.53)	0.65
Systolic blood pressure, mmHg	114 (103, 125)	111 (101, 123)	115 (106, 126)	0.10
Pulse rate, bpm	69 (61, 78)	68 (61, 75)	70 (61, 79)	0.34
Comorbidity				
Atrial fibrillation	26 (12%)	10 (9%)	16 (15%)	0.22
Diabetes mellitus	40 (18%)	23 (21%)	17 (16%)	0.17
Ischemic heart disease	57 (26%)	25 (23%)	32 (29%)	0.21
History of stroke	36 (17%)	17 (16%)	19 (17%)	0.45
History of heart failure admission	91 (42%)	49 (45%)	42 (39%)	0.17
Laboratory data				
Hemoglobin, g/dL	11.0 (10.0, 12.1)	10.9 (10.0, 12.3)	11.0 (9.8, 11.9)	0.45
Serum albumin, g/dL	3.8 (3.5, 4.0)	3.8 (3.6, 4.0)	3.8 (3.5, 4.1)	0.60
Serum sodium, mEq/L	141 (139, 142)	140 (139, 142)	141 (139, 142)	0.66
Serum potassium, mEq/L	4.4 (4.1, 4.6)	4.4 (4.1, 4.7)	4.3 (4.0, 4.6)	0.34
Estimated glomerular filtration ratio, mL/min/m^2^	50 (37, 62)	50 (36, 61)	50 (40, 62)	0.52
Plasma B-type natriuretic peptide, pg/mL	271 (125, 514)	230 (127, 455)	294 (123, 596)	0.31
Echocardiography				
Left ventricular end diastolic diameter, mm	46 (42, 51)	46 (42, 50)	46 (42, 52)	0.93
Left ventricular ejection fraction, %	65 (54, 70)	64 (56, 69)	65 (53, 70)	0.80
Peak velocity at aortic valve, m/s	4.5 (4.0, 4.9)	4.5 (4.0, 4.9)	4.4 (4.1, 4.8)	0.98
Mean pressure gradient at aortic valve, mmHg	47 (38, 57)	46 (38, 58)	47 (39, 57)	0.91
Hemodynamics				
Mean right atrial pressure, mmHg	5 (3, 7)	5 (3, 7)	6 (3, 8)	0.65
Mean pulmonary artery pressure, mmHg	19 (16, 23)	18 (15, 23)	19 (16, 24)	0.27
Pulmonary capillary wedge pressure, mmHg	12 (9, 16)	11 (8, 15)	13 (9, 16)	0.29
Cardiac index, L/min/m^2^	2.7 (2.4, 3.0)	2.6 (2.3, 3.1)	2.7 (2.4, 3.0)	0.70
Medication				
Beta-blocker	71 (33%)	39 (36%)	32 (29%)	0.16
Angiotensin converting enzyme II inhibitor	37 (17%)	16 (15%)	21 (19%)	0.27
Mineralocorticoid receptor antagonist	59 (27%)	33 (31%)	26 (24%)	0.15
Loop diuretics	123 (57%)	63 (58%)	60 (55%)	0.31

Continuous variables are presented as the median and interquartile range and were compared using the Mann–Whitney U test. Categorical variables are presented as a number and percentage and were compared using Fischer’s exact test.

**Table 2:** Impacts of variables on the primary endpoints in the derivation cohort.

	Univariate Analyses		Multivariate Analyses	
	Hazard Ratio (95% Confidence Interval)	*p* Value	Hazard Ratio (95% Confidence Interval)	*p* Value
Continuous variables				
Original SHFM score	0.95 (0.89–1.02)	0.098		
Mean right atrial pressure, mmHg	1.06 (0.86–1.30)	0.57		
Mean pulmonary artery pressure, mmHg	1.05 (0.97–1.13)	0.27		
Pulmonary capillary wedge pressure, mmHg	1.07 (0.99–1.15)	0.052		
Cardiac index, L/min/m^2^	0.19 (0.07–0.52)	0.001 *		
Dichotomized variables				
Original SHFM score < 88.1%	7.99 (1.73–37.0)	0.008 *	5.90 (1.27–27.4)	0.024 *
Mean capillary wedge pressure > 14 mmHg	7.83 (2.08–29.6)	0.002 *	4.70 (1.24–17.9)	0.023 *
Cardiac index < 2.26 L/min/m^2^	6.61 (2.02–21.7)	0.002 *	4.49 (1.36–14.8)	0.014 *
Novel combination variable				
Modified SHFM score	1.38 (1.18–1.61)	<0.001 *		

* *p* < 0.05 by Cox proportional hazard ratio regression analysis. Variables with *p* < 0.10 in the univariate analyses were dichotomized using cutoffs that were calculated using receiver operating characteristics analyses. Dichotomized variables with *p* < 0.05 in the univariate analyses were included in the multivariate analysis.

## Data Availability

Data are available by the corresponding author upon reasonable request.
